# Race and Educational Disparities in Advance Directive Completion: Encouraging Trends in the United States

**DOI:** 10.1093/geroni/igaa057.1061

**Published:** 2020-12-16

**Authors:** Catheryn Koss, Rebecca Hensley

**Affiliations:** California State University, Sacramento, Sacramento, California, United States

## Abstract

Advance directives (AD) help to ensure patients’ wishes are honored and contribute to improved end-of- life care. Race and education disparities in advance directive completion have been extensively documented. This study examined five waves of U.S. Health and Retirement Study exit survey data (N = 7,067) to examine to what extent these disparities have expanded or diminished over the past decade. Overall, advance directive completion increased from about 63% among participants who died in 2005-06 to about 73% among those whose deaths occurred between 2015 and 2016. Non-Hispanic whites were almost four times as likely to have advance directives compared to Hispanics or African Americans across this time period (OR=3.90. p<.0001). However, the growth rate in advance directive completion among non-Hispanic whites was significantly slower than for non-whites (OR=.90, p<.01). Compared to those with a high school education or less, those with some college (OR=1.67, p<.0001) and those with at least a college degree (OR=2.02, p<.0001) were significantly more likely to have advance directives across the time period. There were no significant differences in growth rates of advance directive completion for the different educational categories. These results suggest that educational disparities in advance directive completion are fairly stable, but that race disparities may be diminishing.

